# Identification of a novel fused gene family implicates convergent evolution in eukaryotic calcium signaling

**DOI:** 10.1186/s12864-018-4685-y

**Published:** 2018-04-27

**Authors:** Fei Chen, Liangsheng Zhang, Zhenguo Lin, Zong-Ming Max Cheng

**Affiliations:** 1 State Key Laboratory of Ecological Pest Control for Fujian and Taiwan Crops; Center for Genomics and Biotechnology; Fujian Provincial Key Laboratory of Haixia Applied Plant Systems Biology; Ministry of Education Key Laboratory of Genetics, Breeding and Multiple Utilization of Corps; Fujian Agriculture and Forestry University, Fuzhou, 350002 China; 2 College of Horticulture, Nanjing Agricultural University, Nanjing, 210095 China; 3 Department of Plant Sciences, University of Tennessee, Knoxville, 37996 USA; 4 Department of Biology, Saint Louis University, St. Louis, 63103-2010 USA

**Keywords:** Calcium signaling, Protein phosphorylation, Metakinetoplastina protists

## Abstract

**Background:**

Both calcium signals and protein phosphorylation responses are universal signals in eukaryotic cell signaling. Currently three pathways have been characterized in different eukaryotes converting the Ca^2+^ signals to the protein phosphorylation responses. All these pathways have based mostly on studies in plants and animals.

**Results:**

Based on the exploration of genomes and transcriptomes from all the six eukaryotic supergroups, we report here in Metakinetoplastina protists a novel gene family. This family, with a proposed name *SCAMK*, comprises *SnRK3 fused calmodulin-like III kinase* genes and was likely evolved through the insertion of a *calmodulin-like3* gene into an *SnRK3* gene by unequal crossover of homologous chromosomes in meiosis cell. Its origin dated back to the time intersection at least 450 million-year-ago when Excavata parasites, Vertebrata hosts, and Insecta vectors evolved. We also analyzed *SCAMK*’s unique expression pattern and structure, and proposed it as one of the leading calcium signal conversion pathways in Excavata parasite. These characters made *SCAMK* gene as a potential drug target for treating human African trypanosomiasis.

**Conclusions:**

This report identified a novel gene fusion and dated its precise fusion time in Metakinetoplastina protists. This potential fourth eukaryotic calcium signal conversion pathway complements our current knowledge that convergent evolution occurs in eukaryotic calcium signaling.

**Electronic supplementary material:**

The online version of this article (10.1186/s12864-018-4685-y) contains supplementary material, which is available to authorized users.

## Background

In 1883, animals were first found to use Ca^2+^ as the signaling carrier [1], and in 1910, green plants were also found to rely on Ca^2+^ for plant cell development [2]. Later, cellular and molecular studies identified various types of Ca^2+^ influxes/oscillations known as Ca^2+^ signatures/signals (CS) [3–6] into the eukaryotic cell. Relying on specific types of signal decoding proteins, these CSs are converted into intracellular downstream protein phosphorylation responses (PPRs) [7, 8]. Thereby versatile genes in decoding CSs to PPSs are needed for robust cell signaling.

Up to date, three pathways converting CSs to PPRs have been identified [9, 10]. The type I pathway (Additional file 1: Figure S1) relies on the calmodulin (CaM) for receiving the CSs and convert them to PPRs with interacting kinases such as calcium/calmodulin-dependent protein kinase (CCaMK) [11], calcium /calmodulin-binding protein kinase (CBK) [12], calcium/calmodulin-dependent protein kinase I, II, IV (CAMKI, II, IV) [13]. The type II pathway (Additional file 1: Figure S1) utilizes a single protein calcium-dependent protein kinases (CDPKs) [14] to convert CSs to PPRs. The type III (Additional file 1: Figure S1) employs the calcineurin B-like (CBL) protein to bind the Ca^2+^ and the CBL-interacting protein kinase (CIPK) [15] to convert CSs to PPRs [16, 17]. Based on the fact that CDPK is fused by interacting proteins CaM and CaMK [18], an intriguing yet unknown question is whether there is a convergent gene fusion occurred between CIPK and CBL (Additional file 1: Figure S1), similar to the fusion origin of CDPK.

In eukaryotes, type I CCaMKs exist only in land plants [19] and CaMKIs, IIs, IVs are found in animal and fungi [17, 20], and they also occur in the myxamoeba, *Dictyostelium*. CBKs are found only in plants [21]. Type II CDPK was characterized only in plants and certain protists [22–25]. Type III distributed only in plants and protists *Naegleria gruberi* and *Trichomonas vaginalis* [26]. Although Ca^2+^/CaM regulated protein kinases were also reviewed in *Dictyostelium* and the ciliate, *Paramecium* [27, 28], however, there is still limited analysis of calcium signaling mechanism in other eukaryotic clades Amoebozoa, Excavata, or Stramenopiles-Alveolata-Rizaria (SAR group) [29] compared to the abundant reports in animals and plants.

Here we report a novel fused gene family and date its origin and distribution in metakinetoplastina protists from the Excavata supergroup by mining all the eukaryotic genomes and transcriptomes. We further deduced that such fusion was mediated by an unequal crossover between the homologous chromosomes, yielding an insertion of a *calmodulin-like* (*CML*) *III* gene into the *sucrose non-fermenting related kinase3* (*SnRK3*) kinase gene. We suggest naming this novel type as SCAMK genes. Furthermore, we studied the gene expression pattern, which was highly correlated to [Ca^2+^] changes in different life stages. Finally we proposed that SCAMKs serve as the potential target for drug design in human African trypanosomiasis (HAT).

## Results

### Discovery of a monophyletic gene group with a new structural constitution

**Fig. 1:**
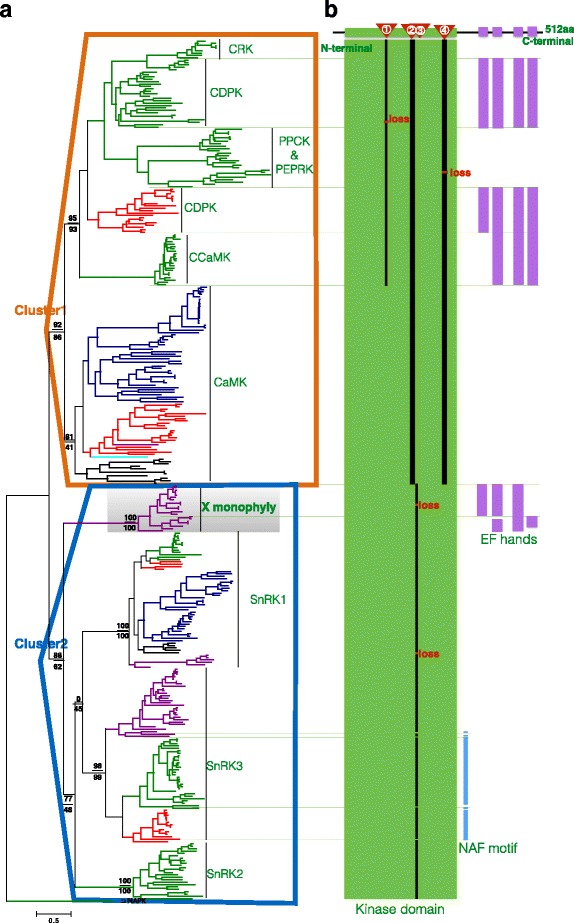
All the Ca^2+^ signal decoders (360 genes) were grouped into two clusters, together with a MAPK gene (outgroup) for phylogenetic tree construction. **a** Phylogenetic relationships among the Ca^2+^ signal decoders identified two monophyletic clusters. Node supporting values of near maximum-likelihood and maximum-likelihood are shown from top to bottom. **b** Conserved sequence insertions (shown as black bars) confirmed the phylogenetic classifications and indicated a new structured monophyly with both N-terminal kinase domain and C-terminal EF hands (shown in purple bars) that were named as the X monophyly genes. Sequences in the tree from Archaeplastida were shown in green, Amoebozoa in black, SAR in red, Opisthokonta in blue, and Excavata in purple

We first set out to identify whether or not there is another kind of Ca^2+^-activated protein kinases by searching all eukaryotic clades based on two criteria, (i) kinome annotations from representatives of five eukaryotic supergroups, *Homo sapiens* [30], *Entamoeba histolytica* [31], *Arabidopsis thaliana* [32], *Leishmania major* [33], *Plasmodium falciparum* [34], and (ii) proteins CDPK, CRK, CCaMK, CIPK with biochemical evidence as the CS decoders [22]. A phylogenetic tree (Fig. 1a) of all the related 360 genes was constructed to show their relationships, with the mitogen-activated protein kinase (MAPK) as the outgroup sequence since it is not regarded as a CS decoder, but closely related to CDPK-SnRK superfamily genes according to all the surveyed kinomes in five supergroups [35, 36]. The complete tree was shown in Additional file 2: Figure S2. Furthermore, we found that all the proteins could be grouped into two monophyletic clusters (Fig. 1a). The cluster I was a well-supported monophyly with a near maximum-likelihood local supporting value (NMLV) 92 using FastTree and a maximum-likelihood bootstrap value (MBV) 86 using RAxML. The cluster I included the CDPKs, CCaMKs and CRKs from both plants and SAR supergroup, together with CaMK I&II&IVs from all eukaryotic supergroups. The cluster II was also a well-supported monophyletic group with an NMLV of 88 and an MBV of 62, which consisted of four subfamilies including three known families SnRK1s, SnRK2s, and SnRK3s. The SnRK3s covered sequences from supergroups Excavata, Arachaeplastida, and SAR. The fourth group from Excavata supergroup contained a kinase domain and EF-handed CaM-like domain. This group had not been reported and we hereby temporarily designated it as the X monophyly.

Since CRKs, CCaMKs, and SnRKs have very different domain structures from CDPKs [22], we compared their protein structures to recheck the phylogenetic classification result. Among all the families, we found conserved sequence insertions supporting our classification (Additional file 3: Figure S3). All the four unique insertions were found in the kinase domain (Fig. 1b). The insertion I had one amino acid (AA), specific to the CRK, CDPK, PPCK, PEPRK, and CCaMK families. Both insertion II and IV had three AAs and they were specific to the cluster I. On the contrary, the insertion III, with one AA, was specific to the cluster II.

At the domain level, the kinase domain (KD) was found in all members in cluster I & II. The CaM-like domain (CaM-LD), which is composed of EF hands, was found in the CDPK, CCaMK families, and the X monophyly (Fig. 1b). SnRK3s from eukaryotic supergroups Excavata, Arachaeplastida, and SAR had the NAF motif, a signature domain of SnRK3 in the C-terminal following the kinase domain, for interaction with the CBL protein [26] (Fig. 1b). However, no exact NAF motif was found in the X monophyletic members.

### Origin of the X monophyly genes in the ancestor of Metakinetoplastina protists

**Fig. 2:**
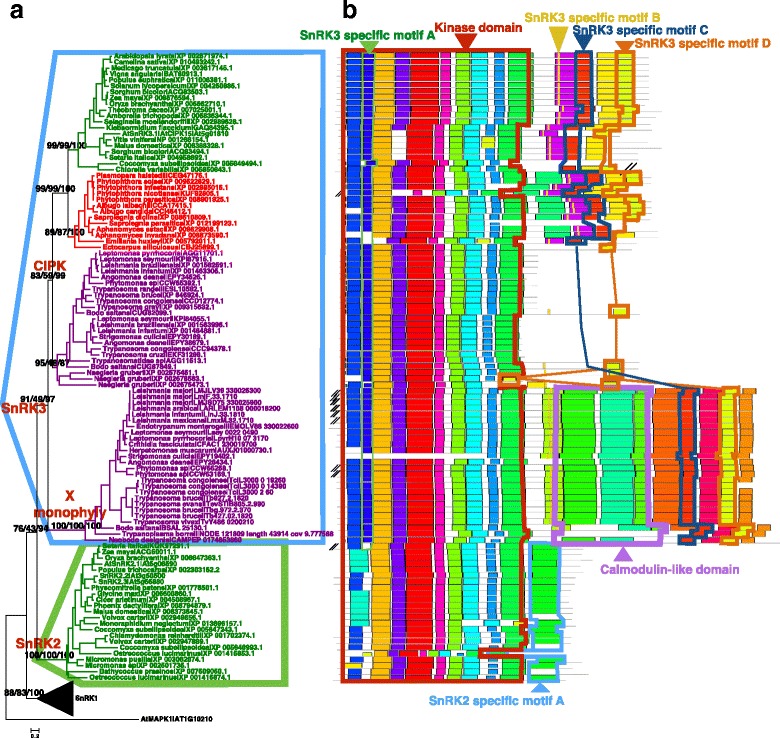
X monophyly genes had a SnRK3 kinase domain and a calmodulin-like domain. **a** Kinase domain-based phylogeny of 130 genes classified X monophyly genes into SnRK3 monophyly. Node supporting values from left to right: near maximum likelihood, maximum likelihood, Bayesian method. **b** SnRK3s had one specific motif in the kinase domain and three specific motifs at the C-terminal, and SnRK2s had one specific motif at the C-terminal

Since these results could not show clearly whether or not the KD of the X monophyly is a member of the SnRK1s/2 s/3 s or a new, fourth subfamily of SnRKs, we then studied the tree phylogeny (Fig. 1) with genome-wide mined X monophyly members (Additional file 4: Table S1), especially two genes in the X monophyly from two basal Metakinetoplastina protists *Trypanoplasma borreli* and *Neobodo designis*. For displaying purpose, we removed a few genes from other subfamilies constructed tree final with 130 genes. We obtained all the representative taxa samples containing the X monophyly genes from the NCBI’s GenBank, genome sequences, and transcriptome sequences (Additional file 4: Table S1). Relying on the KD of the X monophyly genes and all of the other full-length SnRKs, we chose three phylogenetic methods to infer the phylogenetic relationship. In the rooted tree (Fig. 2a) using a MAPK sequence as the outgroup, the X monophyly genes were grouped with the CIPK sequences using all three methods. The Bayesian posterior probability supporting value (BPPV) was notably as high as 97 and Bayesian inference is best for underlying the deep phylogeny. Thirdly, to further validate this phylogeny, we found the motif organizations supported the phylogenetic inference. As shown in Fig. 2b, we found three conserved motifs (motifs were shown as sequence logos in Additional file 5: Figure S4), one in the KD and two specific to the CIPK and the X monophyly genes in the C-terminal. Besides, we identified one motif in the C-terminal specific to the SnRK2s, supporting the improved phylogenetic relationship results in Fig. 2a.

We next investigated the origin of the CaM-LD of the X monophyly genes, to examine our hypothesis that whether it was a CBL, or CaM, or CML, since these three types of four EF-hand proteins are phylogenetically related [37]. We built a tree based on the EF hands of X monophyly genes, CBL, CaM, CML, and the EF hands of CDPK. The whole tree divided CMLs into four subfamilies, CML1-4. The CML IIIs, X monophyly members, and CBLs were closely related (Additional file 6: Figure S5).

**Fig. 3:**
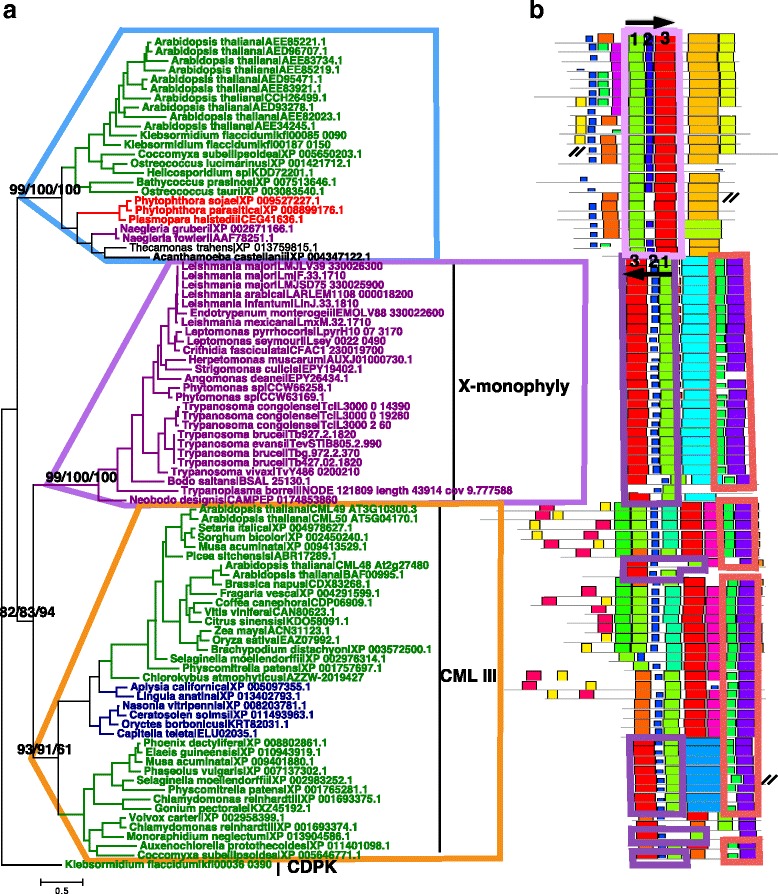
Origin of the calmodulin-like domain (CaM-LD) of the X monophyly genes. **a** The CaM-LD of the X monophyly genes was closely related to the CML IIIs as shown in the phylogenetic tree based on 89 protein sequences. Node supporting values from left to right: near maximum likelihood, maximum likelihood, Bayesian method. **b** The structures of the CaM-LD of the X monophyly genes shared two specific motifs at the C-terminal shown in the red box. The CBLs shared three motifs (in a light purple box) with CMLs, the X monophyly genes (in dark purple box) with a completely reversed order

We further performed combined phylogenetic inference and structural motifs of the three closely related subfamilies CBLs, EF hands of X monophyly, CML IIIs for detailed phylogeny. We found that the X monophyly genes and CML IIIs clustered together with well supported values (NMLV = 82, MLV = 83, BPPV = 94) (Fig. 3a). The CBL was the outgroup to the CML III-X monophyly cluster. Two lines of evidence of gene structural information supported the phylogeny. First, we found two conserved motifs at the C-terminal specific to CML III and the X monophyly. Second, we found three conserved motifs in the middle of the X monophyly proteins that had the same order as those in CML IIIs, but reversed in all CBLs (Fig. 3b).

**Fig. 4:**
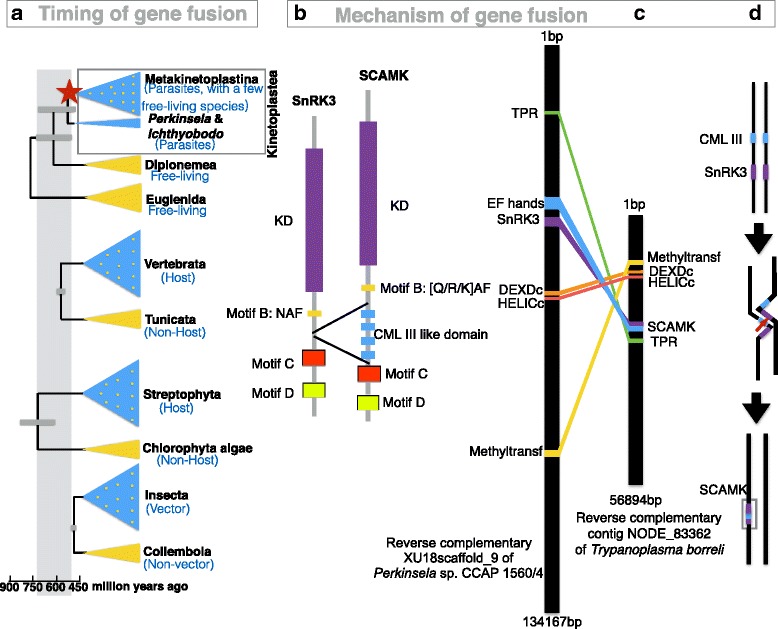
*SCAMK* was proposed for the X monophyly genes based on its origin. **a** The X monophyly genes have been found in Metakinetoplastina protists, and the timing of origin of the parasitic dominant clade Metakinetoplastina coincide with the emergence of diverse hosts including Vertebrata, streptophyta plants, and the Insecta vector. This time period was highlighted in grey block. **b** X monophyly genes originated through the insertion of a calmodulin gene into the C-terminal of a *SnRK3* genes. The insertion site located between the *SnRK3* specific motif B and motif C, thereby we named it as *SnRK3 fused calmodulin-like3 kinase*, *SCAMK*, according to its origin. **c** Gene synteny between the non-*SCAMK* reverse complementary strand of LFNC01000585.1 of *Perkinsela* sp. and the SCAMK-containing reverse complementary contig NODE_83362 of *Trypanoplasma borreli*. **d** The insertion was most likely produced by an unequal crossover of homologous chromosomes

Since the X monophyly genes were present in Metakinetoplastina organisms (subclade of Kinetoplastea) (Fig. 3a), and the genome of *Perkinsela* sp. CCAP 1560/4 (the genus formerly known as *Perkinsiella*) from Prokinetoplastina (subclade of Kinetoplastea) did not contain any X monophyly gene (Additional file 7: Figure S6), it was most likely that the X monophyly genes originated in the ancestor of Metakinetoplastina protists. According to two molecular timing studies based on 15 and 42 protein coding genes, the origin of Metakinetoplastina species occurred ~ 700-450 million-year-ago (mya) [38], and 695-463 mya [39], respectively. Thus, the evolutionary history of the X monophyly genes could be dated back to at least 450 mya. The birth of X monophyly genes coincided roughly with the emergence of hosts streptophytes [40] and vertebrates [38], also coincided with the emergence of vector insects (Fig. 4a) [41].

To explore the mechanism of the origin of X monophyly genes, we hypothesized that they could originate from gene fusion between a SnRK3 kinase and a CML III, which is similar to the origin of CDPK [18]. Since there was no intron in any X monophyly genes (Additional file 4: Table S2), the intron-mediated gene fusion mechanism was ruled out for the birth of the X monophyly genes. Secondly, we also found complete poly-A tail (such as nucleotides 24,774 to 24,779 on the scaffold) after the coding region from basal X monophyly gene from *Trypanoplasma borreli*, suggesting that the X monophyly gene was unlikely to have originated through fusion mediated by transposable elements. The X monophyly genes had one SnRK-specific motif B upstream of the CaM-LD (Fig. 4b), unlike NAF motif found in CIPKs, the cation AA residue N of the NAF motif was changed into anion AA [Q/K/R] in motif B, thereby possibly forbidding its interaction with CaM. Another two SnRK3 specific motifs C & D was found in downstream of the CaM-LD, proving that the CaM-LD in X monophyly genes was inserted into the C-terminal of SnRK3 (Fig. 4b). Therefore, the X monophyly gene was unlikely to have originated by inter-genic chromosome segment loss that resulted in a fusion of upstream and downstream genes.

Because none of the X monophyly gene was present in the genome of *Perkinsela sp*., but present in the genome of *Trypanoplasma borreli*, we further compared the synteny of two genomic blocks from *Perkinsela* sp. and *Trypanoplasma borreli* to show whether the two blocks have evolutionary correlation. We found that the X monophyly gene from *Neobodo designis*, the most basal branch of Metakinetoplastina, had the most related ortholog on the reverse complementary strand of LFNC01000585.1 (Additional file 7: Figure S6) from the genome of *Perkinsela* sp. (Fig. 4c). We also found that four upstream and downstream genes of the X monophyly gene on reverse strand of LFNC01000585.1 from *Perkinsela* sp. and reverse strand contig NODE_83362 from genome of *Trypanoplasma borreli* were conserved syntenic orthologous genes (Fig. 4c). Thus the X monophyly gene might have originated from an unequal crossover between homologous chromosomes in the ancestor of Metakinetoplastina (Fig. 4d). In the crossover stage, the *CML III* gene was inserted into the C-terminal of the kinase gene, leading to the birth of the X monophyly gene.

Considering the X monophyly gene most likely originated from a de novo fusion between an *SnRK3* and a *CML III* gene and without any reported analysis, we propose to name the *X* gene as *SCAMK*, in which ‘CAM’ represents calmodulin-like3 domain, ‘S’ represents SnRK, and ‘K’ represents kinase. The name reflects its insertion evolutionary history.

### Expression profile of the *SCAMK* ortholog from *Trypanosoma brucei*

**Fig. 5:**
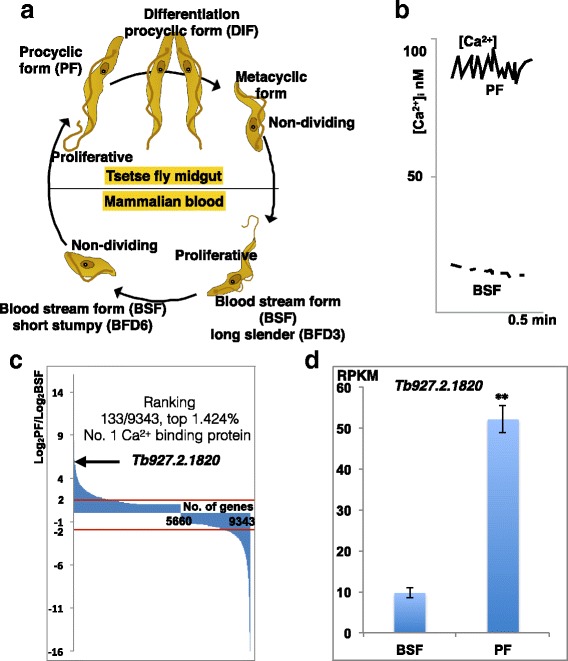
The expression profile of the gene *Tb927.2.1820* from *Trypanosoma brucei*. **a** Life style of *Trypanosoma brucei* is divided into three stages (procyclic form, PF, differentiation procyclic form, DIF, and metacyclic form) in the vector tsetse fly’s midgut, and two stages (blood stream form (BSF) including long slender (could be induced with drug after 3 days) and short stumpy (could be induced after 6 days) in the mammalian blood of the host. **b** Ca^2+^ concentration was reported to be high in the BSF and low in the PF [42]. **c** Expression ratio of whole genome protein coding genes between PF and BSF, with *Tb927.2.1820* marked. **d** Comparison of expressions in the BSF and the PF of the *Trypanosoma brucei*. The unit of the Y-axis is Reads Per Kilobase of transcript per Million mapped reads (RPKM)

To explore the possible molecular activity of the *SCAMK* gene, we studied the expression patterns of a *SCAMK* ortholog in the *Trypanosoma brucei*, a parasitic protozoan causing human African trypanosomiasis that is a neglected tropical disease (www.who.int/neglected_diseases/en/). This unicellular parasite has two main living forms: the procyclic form (PF) in the midgut of the vector tsetse fly (*Glossina* species) and the blood stream form (BSF) in the host human blood (Fig. 5a). In the BSF form, Ca^2+^ concentration was as low as 20-30 nM, but it was up-regulated to about 90 nM in the PF (Fig. 5b) as previously reported [42]. We then measured the expression of all protein coding genes from *T. brucei* between PF and BSF, and found a *SCAMK* ortholog *Tb927.2.1820* expressed significantly higher in the PF than that in the BSF; and it ranked at the top 1.424% among all 9343 genes (Fig. 5c). Notably, *Tb927.2.1820* ranked the highest among all calcium binding protein genes (Additional file 4: Table S3). Specifically, the expression of *Tb927.2.1820* was about 10 Reads Per Kilobase of transcript per Million mapped reads (RPKM) in BSF and increased significantly to ~ 52 RPKM in PF (Fig. 5d), and this increase in expression correlated with the two forms of life styles, as well as with the [Ca^2+^] changes in the cell. This result was further confirmed in a manual induction of the changes of life styles of *Trypanosoma brucei* [43] (Additional file 8: Figure S8)*.* In the cell-dividing BSF stage, the expression was 29 RPKM, and when the cell went into non-dividing short stumpy BSF, the expression dropped significantly to 5 RPKM. In the cell dividing PF, the expression went back to 17 RPKM and reached the peak of 36 RPKM in the cell differentiation procyclic form (DIF) (Additional file 8: Figure S8).

*SCMAK* genes may have potentially significant application. SCAMK proteins had a characteristic domain specific to the SCAMKs in protists (Additional file 9: Figure S9), and such a domain was not found in any of mammal hosts. Thereby, the *TbSCAMK* gene might serve as a potential molecular target for drug design through a protein-ligand docking simulation. It is highly possible to use *TbSCAMK* gene to treat the human African trypanosomiasis (HAT) and related diseases.

## Discussion

### The SCAMK is perhaps the fourth type of CS-PPR converter

**Fig. 6:**
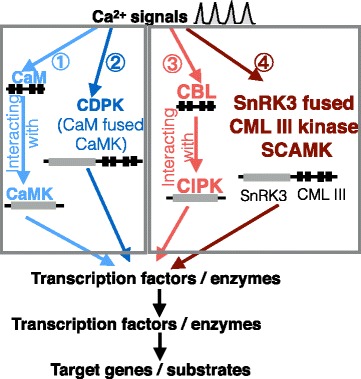
The reported characterized Ca^2+^ signaling pathways and the proposed possible fourth pathway mediated by the SCAMK proteins. The schematic Ca^2+^ wave stands for Ca^2+^ signals. The pathways I and II are shown in bluish lines indicating they are evolutionarily related. The pathways III and IV are shown in reddish lines showing they are evolutionarily related, although the proposed fourth pathway still needs future biochemical validation. The pathway I CCaMK is only found in land plants, and CaMKIs, IIs, IVs are found in animal, fungi, myxamoeba, *Dictiostelium*. The pathway II is found only in plants and certain protists. The pathway III is found in plants and Excavata. The pathway IV is found in Metakinetoplastina protists

Three types of CS decoding pathways [44] mediated by proteins CaMK with interacting CaM, CDPK, and CIPK with interacting CBL have been identified, and they work in two different mechanisms (Fig. 6). *CDPK* was derived from a fusion event of a *CaMK* and *CaM*. It has long been an intriguing evolutionary question whether there is the fourth type of CS decoders, namely a functionally convergent counterpart of CDPK, or, a fusion of CBL and CIPK. Answering such a question would facilitate better understanding of the evolution of CS decoding. In this study, we took advantages of massive genome and transcriptome data from all five supergroups of eukaryotes, and indeed discovered the fused gene by an *SnRK3* gene and a *CML III* gene specifically in Metakinetoplastina protists. We named it as *SCAMK* according to its evolutionary origin. The *SCAMK*s were annotated previously as a *CDPK* gene [33] in GenBank (e.g. www.ncbi.nlm.nih.gov/protein/XP_009310904.1). The kinome-specific database neglected the *SCAMK* genes [45]. In this research, we discovered and proposed that *SCAMK*s originated independently from *CDPK*s by fusion of a *SnRK3* kinase gene and a *CML III* gene, but not with a *CaMK* gene, nor a *CaM*/*CBL* gene. In contrast to *CDPK*, whose fusion was believed to be mediated by the intron [18], *SCAMK* was most likely fused by an unequal crossover of homologous chromosomes.

### The convergently evolved SCAMKs have a conserved evolutionary pattern

This potential fourth type of CS decoder SCAMKs apparently had an independent origin from CDPK. *SCAMK* can be considered as a functional convergently evolved gene similar to *CDPK* and leads to a similar working mechanism by decoding the CS into PPS simultaneously in a single protein as the CDPK does (Additional file 10: Figure S7). The mechanism of the convergent origin of *SCAMK* was rather different from previously known mechanisms in which convergent evolution is mainly caused by AA mutations [46].

Since *SCAMK*s originated in the ancestor of Metakinetoplastina protists, they have maintained their structures and small copy numbers since ~ 450 mya inferred from basal and crown Metakinetoplastina protists. Although Metakinetoplastina protists vary greatly in morphology and in life styles such as free living style (*Bodo* and *Neobodo*), animal parasites (*Leishmania* and *Trypanosoma*), plant parasites (*Phytomona*s), dixenous parasites (=vertebrate or plant host and invertebrate vector) [39], numbers of *SCAMK* genes remain seemingly unchanged. This may be partly due to a lack of genome-wide duplications in Excavata protists [47, 48]. On the other hand, these genes might play vital cellular functional roles and big changes in copy number would lead to the lethal fate.

### The presence of SCAMKs suggests ubiquitous existence of protein phosphorylation following Ca^2+^ binding in Ca^2+^ signaling

The functional convergent evolution of two types of fused CS-PPR converters (CDPK and SCAMK) suggests that evolutionary advantages of eukaryotic cells in utilizing CS to PPR signaling pathways. Prokaryotes rely on two component system (histidine kinase and response regulator protein) for cell signaling [49]. Prokaryotes rely on two component system (histidine kinase and response regulator protein) for cell signaling [49]. In eukaryotes, CDPKs are signaling hub in plant cell signaling [14]. Plants also utilize different combinations of CIPK and CaM for signaling [15]. CaMKI, II, IV and CaM are critical signaling molecules in animals [17, 20]. SCAMKs are active proteins in life form transition of metakinetoplastina protists. These examples show that eukaryotes independently evolved the same mechanism for calcium signaling, i.e. the cooperation of a kinase and a calcium binding protein for signal transduction from calcium signal to protein phosphorylation signals. Specifically, the functional convergent evolution of two types of fused CS-PPR converters (CDPK and SCAMK) suggests that evolutionary advantages of eukaryotic cells in utilizing CS to PPR signaling pathways. Since the emergence of parasitic Metakinetoplastina protists correlates to the emergence of hosts streptophytes and vertebrates, we thereby propose that the transition of free living styles into parasitic living styles might have served as the driving force in leading to the origin of SCAMK, because Ca^2+^ are highly abundant in seawater and terrestrial environments, while eukaryotic cellular Ca^2+^ concentration maintains at very low levels of 100–200 nM [19]. In the future, we could test this hypothesis whether Metakinetoplastina protists could change the life style by knocking out or knocking down the expression of calcium signaling genes such as *SCAMK*s.

### *SCAMK* contributes to trypanosomal cell multiplication and differentiation and illuminates the drug development for HAT and related diseases

Currently, researchers have only identified several proteins that might act as putative drug targets in treating HAT, namely glycogen synthase kinase (GSK) [50], 6-phosphogluconate dehydrogenase (6PGD), proteasome [51], and. However, all these genes are also found in the host human [30, 52] or human gut microbes, and the future drugs should be carefully evaluated for their inhibition to the human or human gut microbes. Other potential drug targets include Dihydrofolate reductase, trypanothione reductase, protein farnesyltransferase, *N*-myristoytransferase, cyclin-dependent kinases, 1,4,5-trisphosphate (IP3) receptor, which are all still being tested as candidates [53–55]. In this report, we found a new family of fusion genes specific to the Metakinetoplastina protists, which may potentially serve as drug targets for HAT. Although the proposed molecules needs biochemical and physiological validation, this potential target site nevertheless provides the ground and first step for future drug development. Similar scenario was proposed for treating malaria: the *Plasmodium* CDPK was proposed as a highly potential drug target in treating malaria [56, 57]. So the comparison of both types of molecular mechanisms would also inspire drug-developing scientists.

Furthermore, the other two neglected tropical diseases listed by the World Health Organization are leishmaniasis and Chagas disease, caused by Excavata protists *Leishmania* and *Trypansosoma cruzi*, respectively. An estimated 900,000-1.3 million new cases and 20,000 to 30,000 deaths of leishmaniasis occur annually. Eight million people estimated to be infected with Chagas disease worldwide, mostly in Latin America (www.who.int/neglected_diseases/en/). In this study, we found that *SCAMK* genes were present in both *Leishmania* spp. and *Trypansosoma* spp.. We also proposed that *SCAMK* genes may be potential molecular drug targets for these diseases based on their unique distribution in these protists, their small copy number, and their potential vital functions in cell signaling. Besides, treatment for leishmaniasis is limited because the currently available drugs vary greatly in efficacy depending on the infecting *Leishmania* spp. [58]. Meanwhile, trypanotolerance distributed widely in human and animals [59]. We have shown in this study that *SCAMK*s were very conserved both in structures and in numbers among *Leishmania* and *Trypansosoma* spp.. Considering its very conserved evolutionary pattern, we believed that SCAMKs are very promising candidate targets for treating diseases by *Leishmania* and *Trypansosoma* spp., as it has proved that genomics can lead to the development of treatments for these neglected tropical diseases today and in the future [60].

## Methods

### Datasets and sequence retrieval

Ca^2+^/calmodulin-dependent protein kinase (CAMK) sequences from the kinomes of *Arabidopsis thaliana* (supergroup Archaeplastida), *Trypanosoma brucei* (supergroup Excavata), *Plasmodium falciparum* (supergroup SAR), *Homo sapiens* (supergroup Opisthokonta), and *Entamoeba histolytica* (supergroup Amoebozoa) were retrieved from curated databases (Additional file 4: Table S1). They were combined as the seed for hidden Markov model based search using HMMER software [61]. Data resources from Excavata species were retrieved from several public databases enclosed in Additional file 4: Table S1. The other CAMK sequences from plants, animals, and fungi were obtained using BLAST search against the NCBI database. All the sequence IDs were listed in the tree for clarity.

### Sequence alignment and phylogenetic tree construction

Only protein sequences were used to infer the sequence evolution since they are more neutral than the DNA as we traced the origin of SCAMK genes to be as old as 0.4 billion year ago. Sequences were aligned using online tool mafft (www.ebi.ac.uk/Tools/msa/mafft/), which performs well with large dataset [62]. No manual adjustment were made to all the alignments. Near Maximum-likelihood phylogenetic tree was constructed by FastTree [63]. Maximum-likelihood phylogenetic tree was constructed by RAxML software [64] with 1000 bootstrap samplings. Both RAxML and FastTree methods were used for tree construction in each figure. Bayesian phylogenetic tree was constructed using Mrbayes [65].

### Gene, domain, motif, protein predictions

Genes on the scaffolds were predicted using Genescan [66]. Protein domains were predicted by searching against both the SMART domain database and the Pfam domain database using SMART software [67]. Motifs were predicted by MEME (meme-suite.org). The three-dimensional structure of the SCAMK protein was de novo modeled using the online I-TASSER server [68]. Protein-ligand docking was modeled relying on online server SwissDock [69].

### Expression calculation

The transcriptome sequences and the expression value of all the protein-coding genes *Trypanosoma brucei* were obtained from reported projects [43, 70], which were both based on paired-end Illumina sequencing. We mapped and quantified expression values using reads per kilobase per million mapped reads (RPKM) method. Average expression values of 9343 genes among three biological replicates were calculated both at procyclic form and blood stream form of *T. brucei*. We tested for significant difference using Duncan’s new multiple range test implemented in the SPSS software [71].

## Conclusions

The critical role that Ca^2+^ signaling played in many subcellular processes have been well established and known in plants and animals, whereas the role about protozoa is largely restricted. Relied on recent advances in genome and transcriptome development, this report identified a novel gene fusion and dated its precise fusion time in Metakinetoplastina protists. The fused gene family was termed as SCAMK based on its gene insertion history. Its copy number and expression pattern was studied in the parasite protist for the first time. This potential fourth eukaryotic calcium signal conversion pathway complements our current knowledge that convergent evolution occurs in eukaryotic calcium signaling.

## Additional files

Additional file 1: **Figure S1.** SCAMK proteins mediate three calcium signal decoding pathways and the hypothesized fourth one in this research. (PDF 38 kb)

Additional file 2: **Figure S2.** The complete phylogenetic tree displaying two monophyly clusters in Fig. 2. Supporting values on the tree were produced by FastTree. Sequences from plant were shown in green, Amoebozoa in black, SAR in red, Opisthokonta in blue, and Excavata in purple. (PDF 73 kb)

Additional file 3: **Figure S3.** The four insertions in the kinase domain found in Fig. 1 were shown in sequence logo format. (PDF 376 kb)

Additional file 4: **Table S1.** Samples and data resources used in this research. **Table S2.** Characteristics of *SCAMK* genes. **Table S3.** Top 133 up-regulated genes in PF of *T. brucei*. Tb927.2.1820 was highlighted in green. (DOCX 450 kb)

Additional file 5: **Figure S4.** Five specific motifs found in Fig. 3 were shown in sequence logo format. (PDF 1561 kb)

Additional file 6: **Figure S5.** Phylogenetic relationships among CaMs, CMLs, CBLs, CDPKs, and X monophyly members. (PDF 43 kb)

Additional file 7: **Figure S6.** Scaffold of the genome released *Perkinsela* sp. has a most realted homolog to the X monophyly gene member *CAMPEP_0174853860* from *Neobodo designis*. (PDF 555 kb)

Additional file 8: **Figure S8.** The expression changes among two stages in the BSF cells grown for 3 days (BFD3) and 6 days (BFD6), and two stages (PF and DIF) in the PF, together with a non-tetracycline (no_Tet) induction form as the control. Two stars indicated significance at *P* ≤ 0.01. The raw expression data were from reported projects [43, 70]. (PDF 35 kb)

Additional file 9: **Figure S9.** A SCAMK-specific motif could serve as the target domain for drug design as validated by a barb scaffold molecule docking to the target domain of Tb927.2.1820 protein*.* (A) Phylogenetic tree showing the SCAMKs in metakinetoplastina and the SnRK1s in the Metazoa including host and vector. (B) Compared to the SnRK1s, SCAMKs have a specific calmodulin-like domain and a target domain. (C) A barb scaffold molecule was specifically docked to the target domain of Tb927.2.1820 protein with high affinity (barb molecule structure from the drug-like ligand small molecule database SwissDock. (PDF 863 kb)

Additional file 10: **Figure S7.** *Perkinsela* sp. did not contain any X monophyly member. (PDF 237 kb)

## Abbreviations

6PGD: 6-phosphogluconate dehydrogenase
AA: Amino acid
BPPV: Bayesian posterior probability supporting value
BSF: Blood stream form
CaM: Calmodulin
CaMK I/II/IV: Calcium/calmodulin-dependent protein kinase I/II/IV
CaM-LD: Calmodulin-like domain
CBK: Calcium /calmodulin-binding protein kinase
CBL: Calcineurin B-like
CCaMK: Calcium/calmodulin-dependent protein kinase
CDPK: Calcium dependent protein kinase
CIPK: CBL-interacting protein kinase
CML: Calmodulin-like
CS: Ca^2+^ signatures/signal
GSK: Glycogen synthase kinase
HAT: Human African trypanosomiasis
IP3: 1,4,5-trisphosphate
MAPK: Mitogen-activated protein kinase
MBV: Maximum-likelihood bootstrap value
NMLV: Near maximum-likelihood local supporting value
PF: Procyclic form
PPRs: Protein phosphorylation responses
RPKM: Reads per kilobase per million mapped reads
SAR: Stramenopiles-Alveolata-Rizaria
SCAMK: SnRK3 and CML III fused kinase
SnRK: Sucrose non-fermenting related kinase
