# Supplementary material for: Identification of a novel fused gene family implicates convergent evolution in eukaryotic calcium signaling
Source: BMC Genomics. 2018 Apr 27;19:306. doi: 10.1186/s12864-018-4685-y (PMC5924475; doi:10.1186/s12864-018-4685-y)
Supplement: Supplementary file 1 — Figure S1. SCAMK proteins mediate three calcium signal decoding pathways and the hypothesized fourth one in this research. (PDF 38 kb) [file 12864_2018_4685_MOESM1_ESM.pdf]

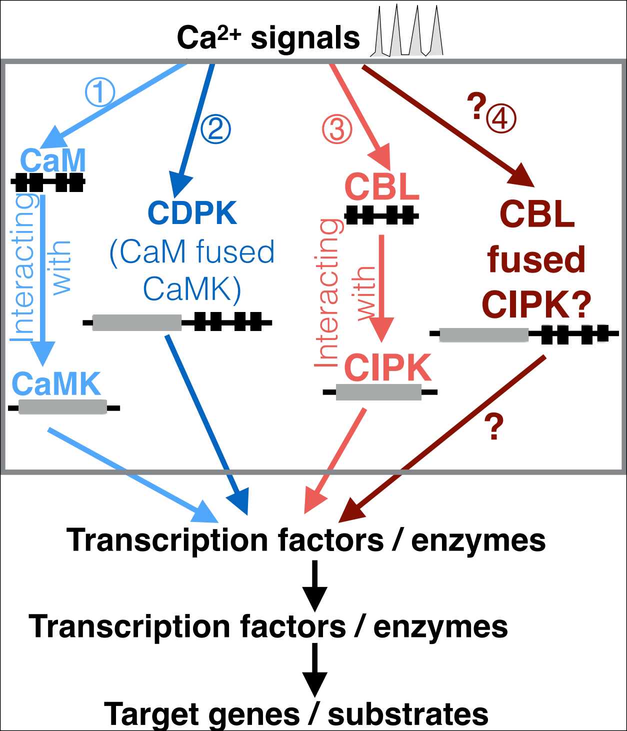

Ca²⁺ signals
①
CaM
Interacting with
CaMK
②
CDPK
(CaM fused CaMK)
③
CBL
Interacting with
CIPK
?④
CBL fused CIPK?
?
Transcription factors / enzymes
Transcription factors / enzymes
Target genes / substrates
