# Supplementary figures and images for: Identification of a novel fused gene family implicates convergent evolution in eukaryotic calcium signaling
Source: BMC Genomics. 2018 Apr 27;19:306. doi: 10.1186/s12864-018-4685-y (PMC5924475; doi:10.1186/s12864-018-4685-y)

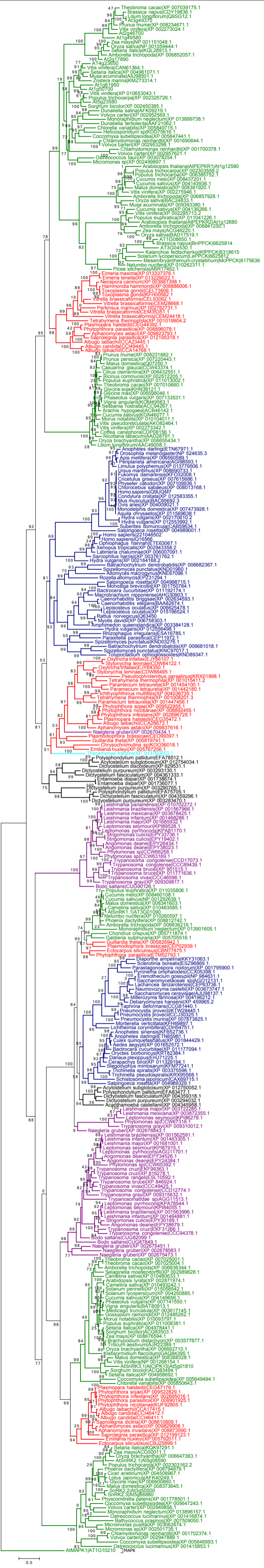

Supplement: Supplementary file 2 — Figure S2. The complete phylogenetic tree displaying two monophyly clusters in Fig. 2. Supporting values on the tree were produced by FastTree. Sequences from plant were shown in green, Amoebozoa in black, SAR in red, Opisthokonta in blue, and Excavata in purple. (PDF 73 kb) [file 12864_2018_4685_MOESM2_ESM.pdf]

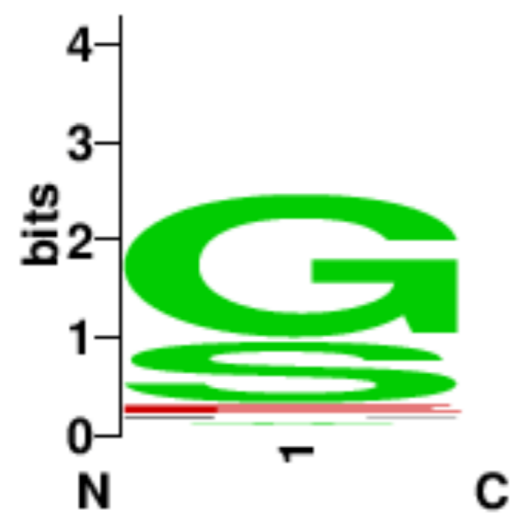

Insertion1

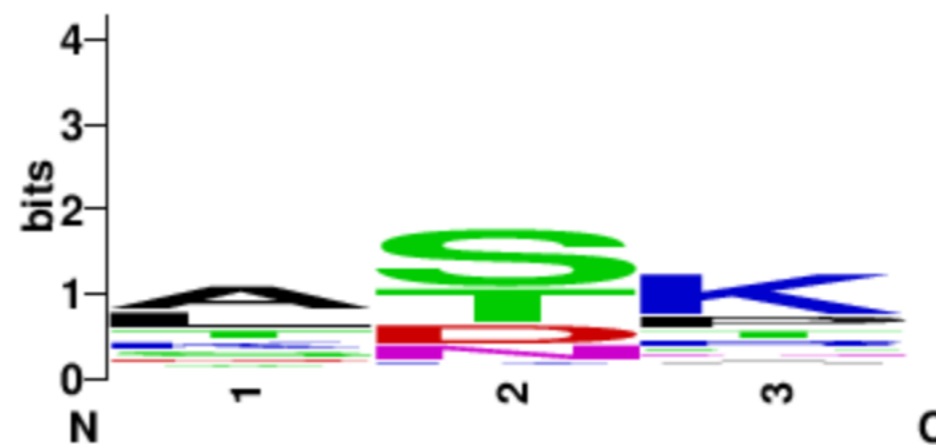

Insertion2

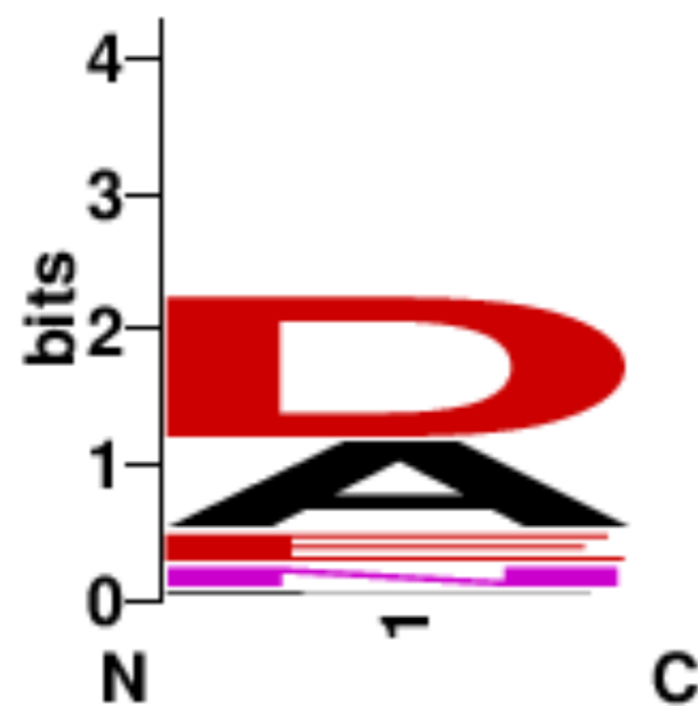

Insertion3

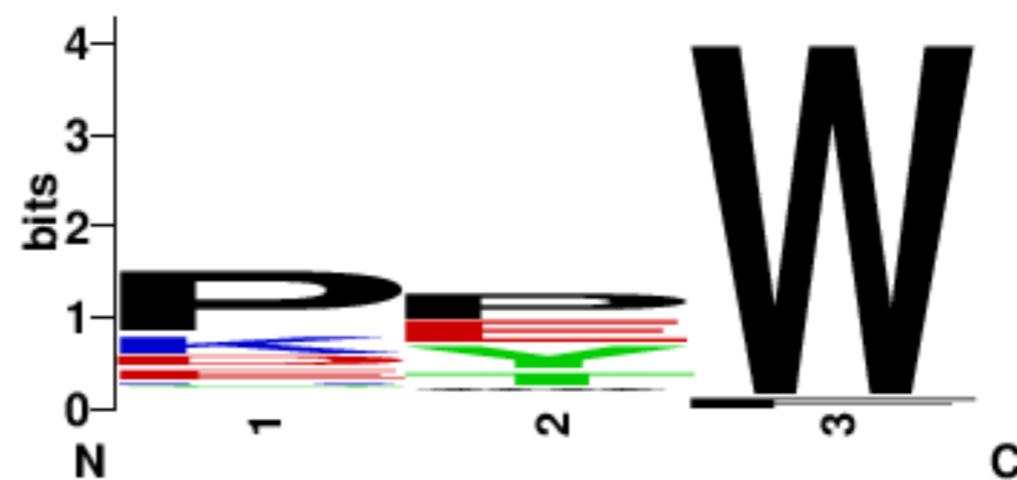

Insertion4

Supplement: Supplementary file 3 — Figure S3. The four insertions in the kinase domain found in Fig. 1 were shown in sequence logo format. (PDF 376 kb) [file 12864_2018_4685_MOESM3_ESM.pdf]

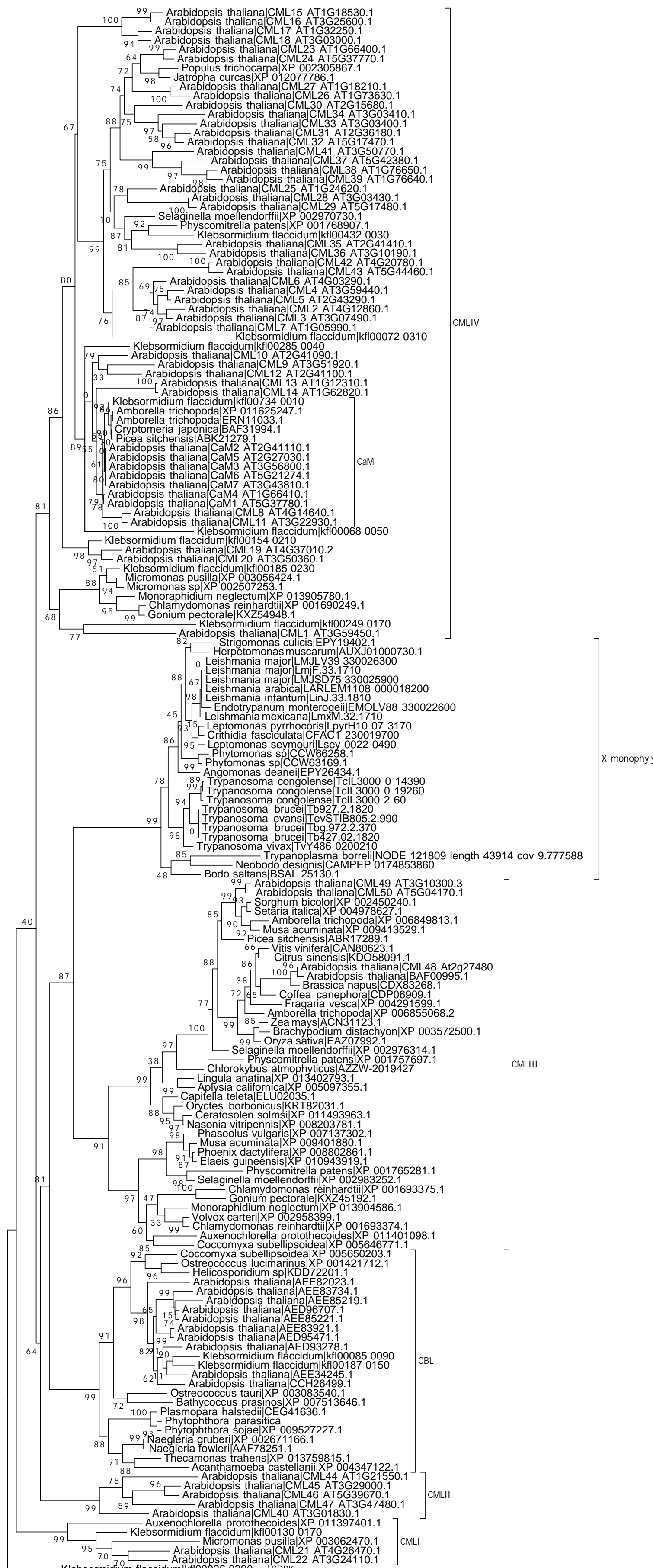

Supplement: Supplementary file 6 — Figure S5. Phylogenetic relationships among CaMs, CMLs, CBLs, CDPKs, and X monophyly members. (PDF 43 kb) [file 12864_2018_4685_MOESM6_ESM.pdf]

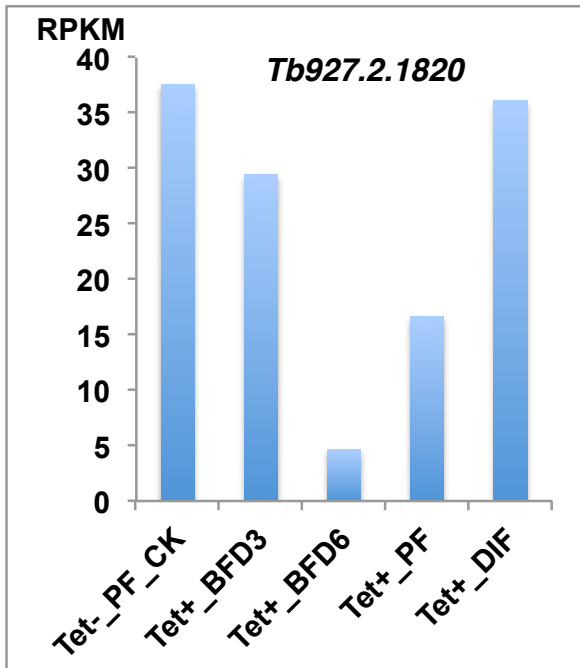

Supplement: Supplementary file 8 — Figure S8. The expression changes among two stages in the BSF cells grown for 3 days (BFD3) and 6 days (BFD6), and two stages (PF and DIF) in the PF, together with a non-tetracycline (no_Tet) induction form as the control. Two stars indicated significance at P ≤ 0.01. The raw expression data were from reported projects [43, 70]. (PDF 35 kb) [file 12864_2018_4685_MOESM8_ESM.pdf]

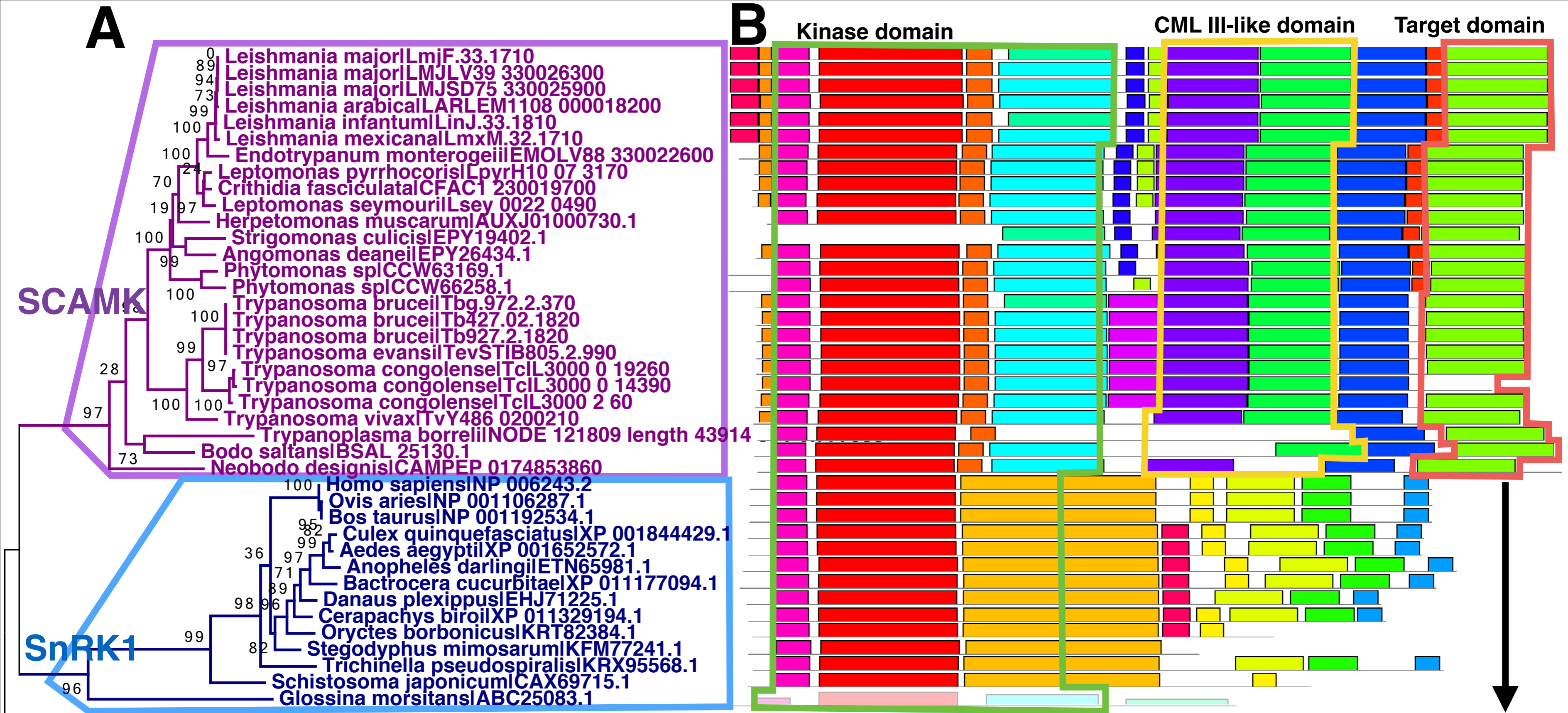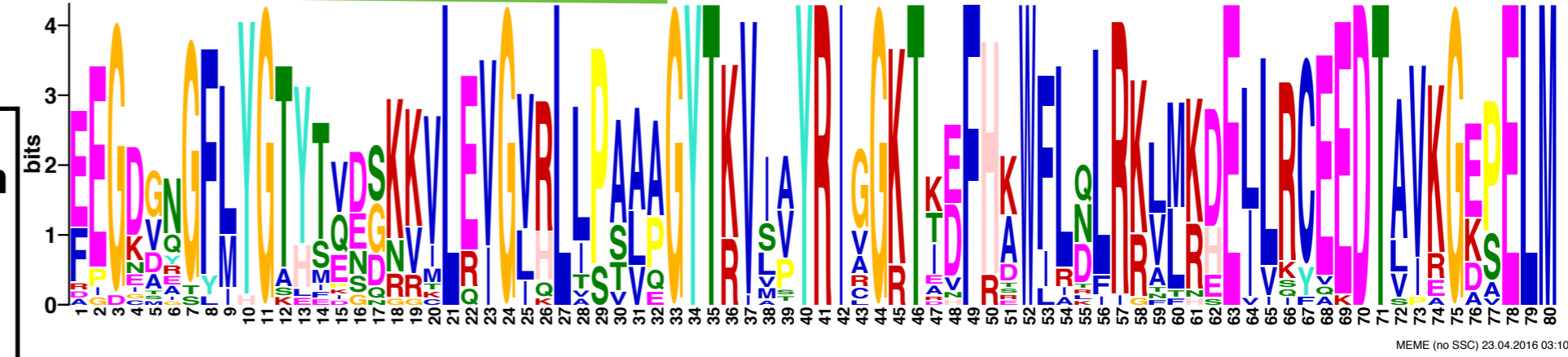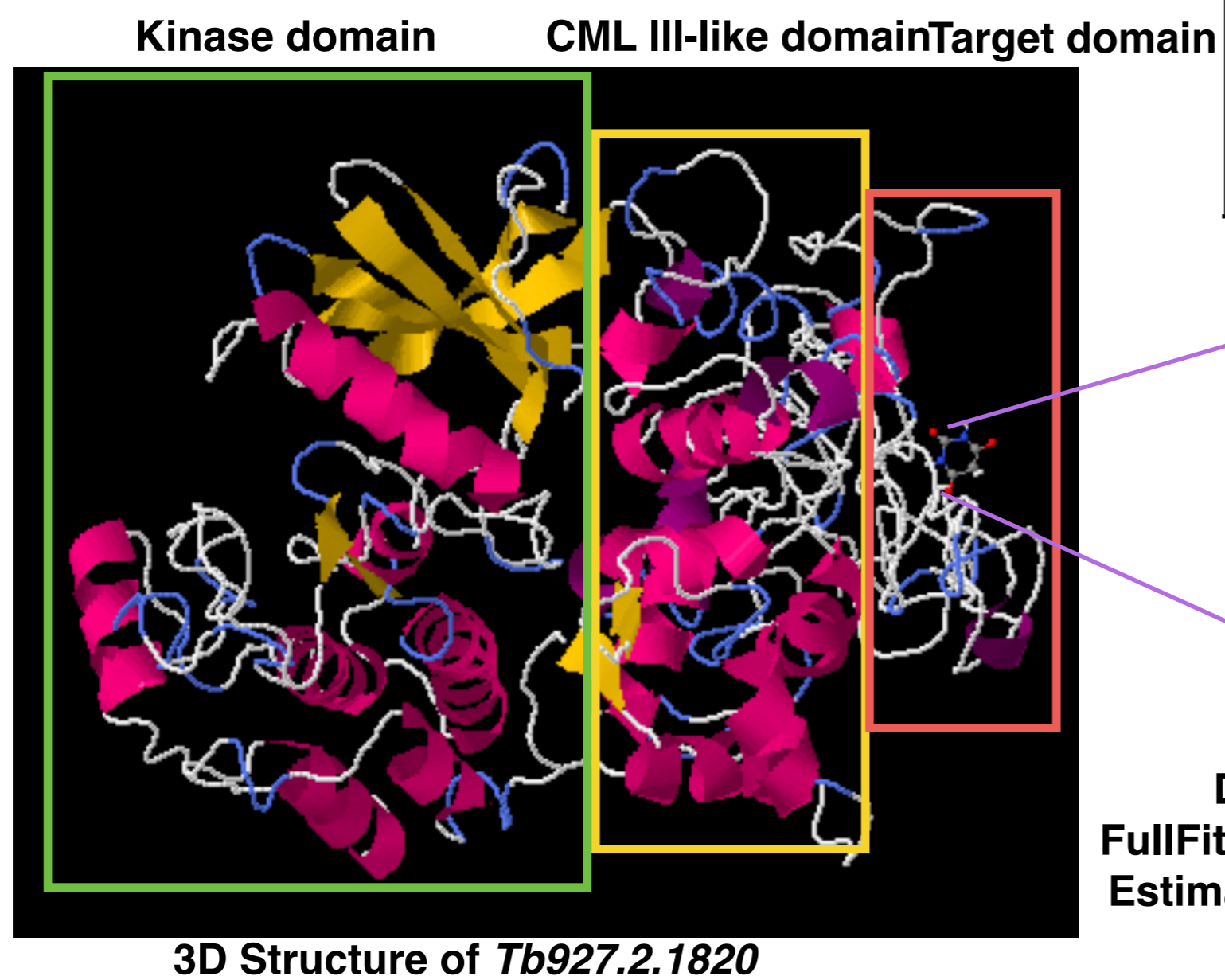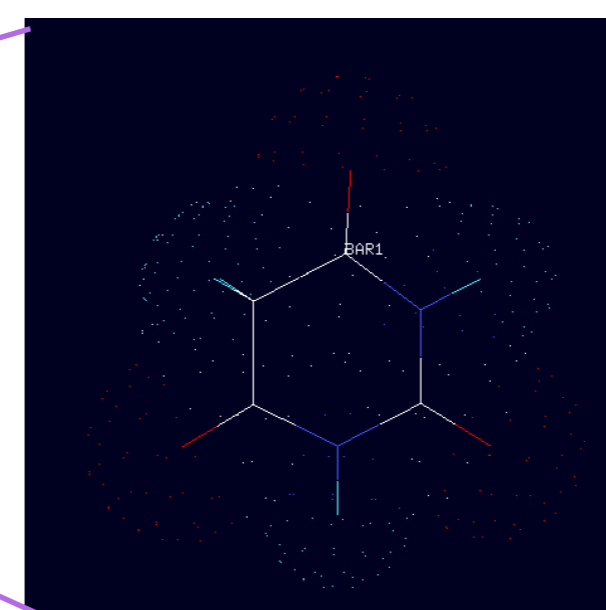

**Docking parameter:**  
FullFitness (kcal/mol)=-2326.87  
Estimated  $\Delta G$  (kcal/mol)=-5.69

Supplement: Supplementary file 9 — Figure S9. A SCAMK-specific motif could serve as the target domain for drug design as validated by a barb scaffold molecule docking to the target domain of Tb927.2.1820 protein. (A) Phylogenetic tree showing the SCAMKs in metakinetoplastina and the SnRK1s in the Metazoa including host and vector. (B) Compared to the SnRK1s, SCAMKs have a specific calmodulin-like domain and a target domain. (C) A barb scaffold molecule was specifically docked to the target domain of Tb927.2.1820 protein with high affinity (barb molecule structure from the drug-like ligand small molecule database SwissDock. (PDF 863 kb) [file 12864_2018_4685_MOESM9_ESM.pdf]

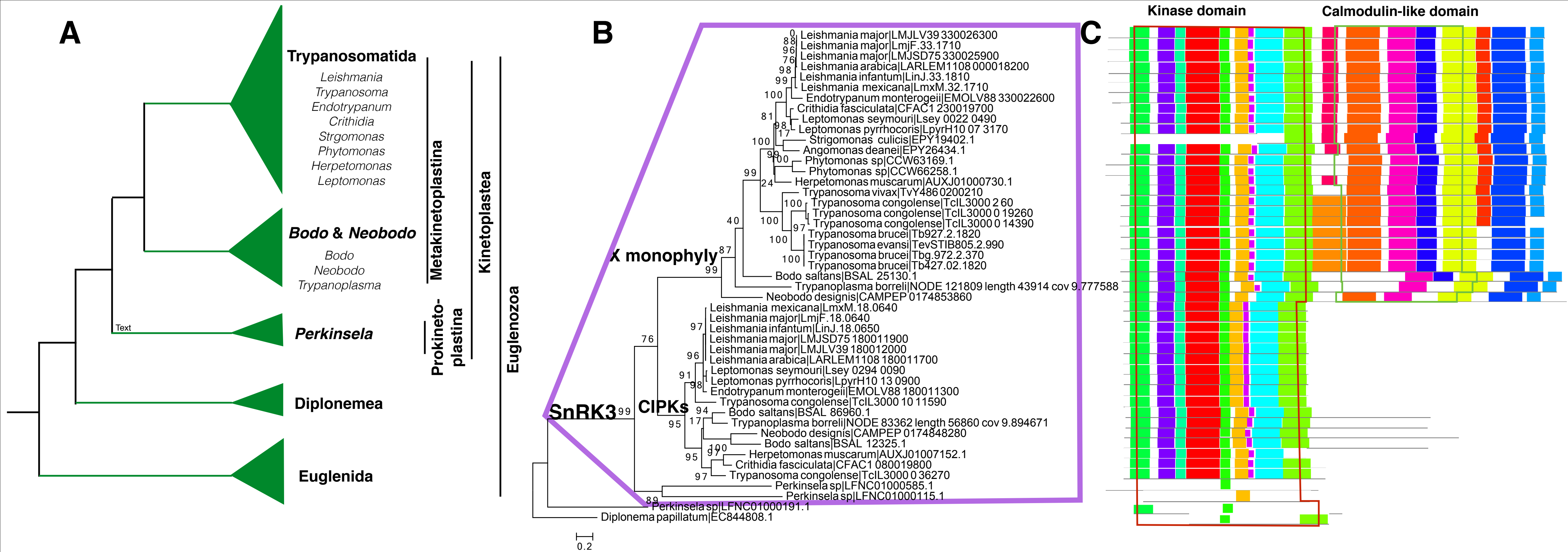

Supplement: Supplementary file 10 — Figure S7. Perkinsela sp. did not contain any X monophyly member. (PDF 237 kb) [file 12864_2018_4685_MOESM10_ESM.pdf]
