# Supplementary material for: Identification of a novel fused gene family implicates convergent evolution in eukaryotic calcium signaling
Source: BMC Genomics. 2018 Apr 27;19:306. doi: 10.1186/s12864-018-4685-y (PMC5924475; doi:10.1186/s12864-018-4685-y)
Supplement: Supplementary file 4 — Table S1. Samples and data resources used in this research. Table S2. Characteristics of SCAMK genes. Table S3. Top 133 up-regulated genes in PF of T. brucei. Tb927.2.1820 was highlighted in green. (DOCX 450 kb) [file 12864_2018_4685_MOESM4_ESM.docx]

**Supplementary Information**

**Table S1.** Samples and data resources used in this research.

**Table S2.** Characteristics of *SCAMK* genes.

| Notes: *N-terminal modifications include N-myristoylation and N-palmytoylation. |
| --- |
| N-terminal N-myristoylation ofProteins: http://mendel.imp.ac.at/myristate/SUPLpredictor.htm |
| N-terminal palmytoylation prediction: http://csspalm.biocuckoo.org/online.php |
| PEST motif,http://emboss.bioinformatics.nl/cgi-bin/emboss/epestfind |
| ** predicted by WoLF PSORT (www.genscript.com/wolf-psort.html) with the synthesized results from animals, plants, fungi |
| cyto: cytosol; extr: Secreted; NA: undetermined; null: none predicted |

**Table S3.** Top 133 up-regulated genes in PF of *T. brucei*. Tb927.2.1820 was highlighted in green.
