# Supplementary material for: Identification of a novel fused gene family implicates convergent evolution in eukaryotic calcium signaling
Source: BMC Genomics. 2018 Apr 27;19:306. doi: 10.1186/s12864-018-4685-y (PMC5924475; doi:10.1186/s12864-018-4685-y)
Supplement: Supplementary file 7 — Figure S6. Scaffold of the genome released Perkinsela sp. has a most realted homolog to the X monophyly gene member CAMPEP_0174853860 from Neobodo designis. (PDF 555 kb) [file 12864_2018_4685_MOESM7_ESM.pdf]

Neobodo_designis|CAMPEP_0174853860

RID 5Y9KSEAA015 (Expires on 12-04 00:12 am)

Query ID lcl|Query_55187
Description Neobodo_designis|CAMPEP_0174853860
Molecule type amino acid
Query Length 650

Database Name WGS_VDB://LFNC01
Description
Program TBLASTN 2.3.0+ Citation

Other reports: Search Summary

Graphic Summary

Distribution of 32 Blast Hits on the Query Sequence

Mouse-over to show defline and scores, click to show alignments

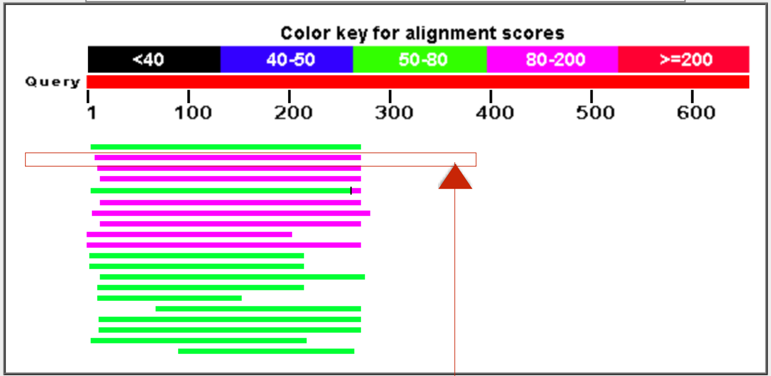

Alignments Download GenBank Graphics

	Description	Max score	Total score	Query cover	E value	Ident	Accession
	Perkinsela sp. CCAP 1560/4 XU18scaffold_1, whole genome shotgun sequence	124	405	40%	1e-29	31%	LFNC01000001.1
	Perkinsela sp. CCAP 1560/4 XU18scaffold_9, whole genome shotgun sequence	114	114	40%	2e-26	29%	LFNC01000585.1
	Perkinsela sp. CCAP 1560/4 XU18scaffold_2, whole genome shotgun sequence	113	321	39%	4e-26	31%	LFNC01000115.1
	Perkinsela sp. CCAP 1560/4 XU18scaffold_137, whole genome shotgun sequence	104	104	39%	2e-23	30%	LFNC01000079.1
	Perkinsela sp. CCAP 1560/4 XU18scaffold_29, whole genome shotgun sequence	101	177	40%	2e-22	29%	LFNC01000169.1
	Perkinsela sp. CCAP 1560/4 XU18scaffold_74, whole genome shotgun sequence	100	149	39%	3e-22	29%	LFNC01000462.1
	Perkinsela sp. CCAP 1560/4 XU18scaffold_32, whole genome shotgun sequence	93.2	131	42%	8e-20	24%	LFNC01000191.1
	Perkinsela sp. CCAP 1560/4 XU18scaffold_7, whole genome shotgun sequence	88.2	88.2	39%	3e-18	23%	LFNC01000426.1
	Perkinsela sp. CCAP 1560/4 XU18scaffold_15, whole genome shotgun sequence	86.7	86.7	31%	7e-18	28%	LFNC01000087.1
	Perkinsela sp. CCAP 1560/4 XU18scaffold_8, whole genome shotgun sequence	85.5	85.5	41%	2e-17	28%	LFNC01000508.1
	Perkinsela sp. CCAP 1560/4 XU18scaffold_209, whole genome shotgun sequence	78.2	78.2	32%	3e-15	28%	LFNC01000124.1
