# Supplementary material for: Identification of a novel fused gene family implicates convergent evolution in eukaryotic calcium signaling
Source: BMC Genomics. 2018 Apr 27;19:306. doi: 10.1186/s12864-018-4685-y (PMC5924475; doi:10.1186/s12864-018-4685-y)
Supplement: Supplementary file 5 — Figure S4. Five specific motifs found in Fig. 3 were shown in sequence logo format. (PDF 1561 kb) [file 12864_2018_4685_MOESM5_ESM.pdf]

Motif	Sequence logo	E-value
SnRK3 specific motif A	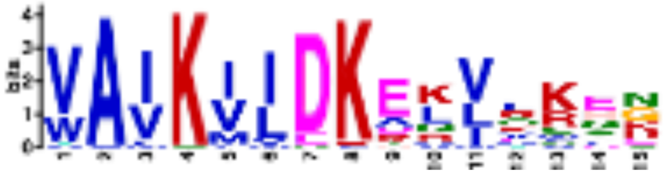	3.2e-480
SnRK3 specific motif B	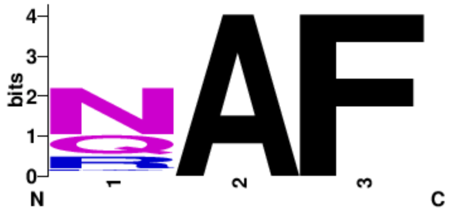	NA
SnRK3 specific motif C	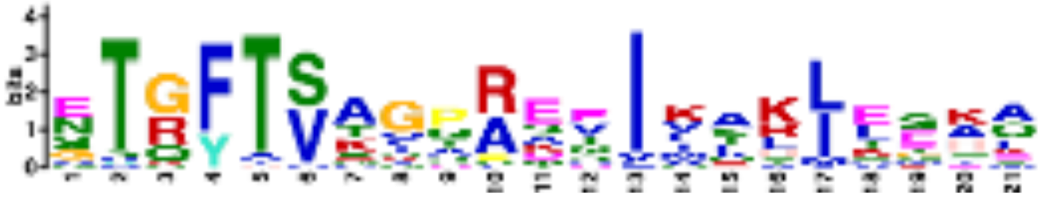	9.6e-329
SnRK3 specific motif D	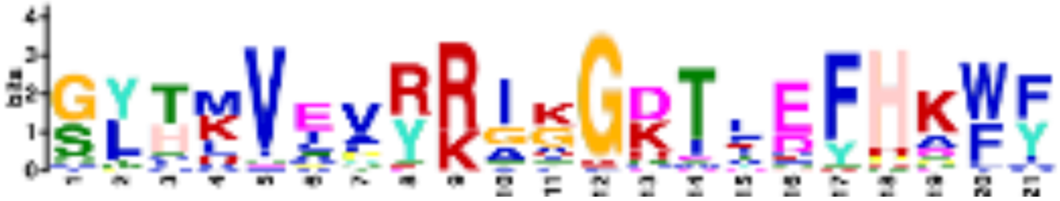	1.4e-536
SnRK2 specific motif A	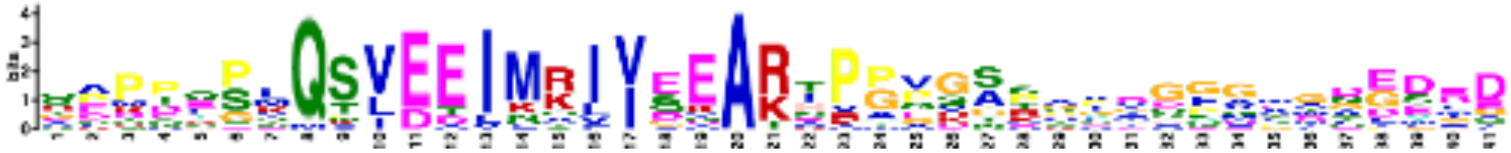	7.5e-141
